# Grasping the Changes Seen in Older Adults When Reaching for Objects of Varied Texture

**DOI:** 10.1371/journal.pone.0069040

**Published:** 2013-07-31

**Authors:** Raymond J. Holt, Alexis S. Lefevre, Ian J. Flatters, Pete Culmer, Richard M. Wilkie, Brian W. Henson, Geoff P. Bingham, Mark Mon-Williams

**Affiliations:** 1 School of Mechanical Engineering, University of Leeds, Leeds, United Kingdom; 2 Institute of Psychological Sciences, University of Leeds, Leeds, United Kingdom; 3 Department of Psychological and Brain Sciences, Indiana University, Bloomington, Indiana, United States of America; Emory University, United States of America

## Abstract

Old age is associated with reduced mobility of the hand. To investigate age related decline when reaching-to-lift an object we used sophisticated kinematic apparatus to record reaches carried out by healthy older and younger participants. Three objects of different widths were placed at three different distances, with objects having either a high or low friction surface (i.e. rough or slippery). Older participants showed quantitative differences to their younger counterparts – movements were slower and peak speed did not scale with object distance. There were also qualitative differences with older adults showing a greater propensity to stop the hand and adjust finger position before lifting objects. The older participants particularly struggled to lift wide slippery objects, apparently due to an inability to manipulate their grasp to provide the level of precision necessary to functionally enclose the object. These data shed light on the nature of age related changes in reaching-to-grasp movements and establish a powerful technique for exploring how different product designs will impact on prehensile behavior.

## Introduction

Reaching-to-grasp-and-lift an object underpins many activities of daily living. There has been little systematic study of this behavior in older adults. This is deeply disappointing as many older adults have problems safely grasping objects and an increased understanding of the underlying difficulties might allow the manufacture of objects better suited to older hands. Reaching-and-grasping an object is integral to many ‘Activities of Daily Living’ [1,2]. Conditions such as stroke and arthritis have an obvious impact on grasping but even healthy aging reduces hand function and this can limit independence [3,4]. Grip strength declines with age [5–7] though older adults often apply greater force to grasp an object [8]. These findings raise questions regarding the movements generated by older adults as they reach to grasp objects – i.e. before the point at which forces are applied to objects.

The pre-contact phase of reaching-to-grasp involves two important actions: targeting (positioning the fingertips in the right place to grasp the object [9]) and collision avoidance (ensuring that the fingers do not knock the object over while being positioned [10]). The precise configuration of the reach-to-grasp behaviour is tailored to the object: larger objects elicit palmar grasps and smaller objects produce precision grips [11]. The precision grip [11] describes grasping an object between forefinger and thumb and is central to many activities of daily living (e.g. picking up a pen). Reaching-to-grasp with a precision grip shows stereotypical patterns in young neurologically intact adults. The hand accelerates to a peak speed, with the forefinger and thumb separating to create a ‘grip aperture’. Peak speed is scaled to the distance of the object, with higher peak speed associated with further objects. The hand then decelerates as it approaches the object at which point the grip aperture reaches its maximum and begins to close. Maximum grip aperture is generally scaled to object size, such that it is always slightly larger than the object itself (allowing the fingers to close around the object rather than colliding with it), and tends to occur later in the movement for larger objects [12–15]. Mon-Williams and Bingham [16] showed that the physical properties of an object influence the qualitative spatial structure of reach-to-grasp motions and identified two distinct strategies to grasp an object: a ‘stop’ motion, where the hand reaches the object then pauses to position the fingers; and a ‘fly-through’ movement where the hand reaches and grasps the object without a pause.

Flatters et al. [17], investigated the strategies identified by Mon-Williams and Bingham [16] in order to explore the effect of object texture on reach-to-grasp behaviour. In the Flatters et al. [17], study, participants reached-to-grasp objects with three different surface textures. Texture was manipulated because different coefficients of friction alter the safety margins of reach-to-grasp tasks. Manually securing an object requires the frictional force to be greater than the tangential component of object weight at the interface between the digits and the object [17]. A curved grasp surface results in a ‘cone of friction’ specifying the spatial zone in which the digits must apply force for a stable grasp. A decrease in the coefficient of friction causes a reduction in the size of the zone. Thus, lower friction surfaces decrease the safety margins of the task. It should also be noted that increasing the width of the object decreases the safety margins because of the anatomical limit of how wide the hand can open (the maximum grip aperture [16]). In line with this analysis of task constraints, Flatters et al. [17], showed that a low friction (slippery) surface almost inevitably caused participants to stop their hand moving forward before an object was secured between the index finger and the thumb and then lifted from the tabletop. In other words, the proportion of fly through movements was reduced when the coefficient of friction decreased. Likewise, Mon-Williams and Bingham [16] found that increasing the width of the objects caused a decrease in the proportion of fly through movements. Flatters et al. [17], found that width and friction coefficient interacted such that the wider the object the more difficult it is to grasp as the coefficient of friction decreased.

Mon-Williams and Bingham [16] related the safety margins of the reach-to-grasp task to hand size. This relationship was derived within the theoretical framework of ‘affordance perception’ [18]. Affordances can be defined as are dispositional properties that relate the perspective of the relevant action to the corresponding properties of the actor [19]. Mon-Williams and Bingham [16] argued that the relevant property of the actor in a reach-to-grasp task is the opposition axis or vector. Iberall et al. [20], introduced the ‘opposition axis’ as a unit of analysis for reach-to-grasp actions. The axis extends between the opposing thumb and finger(s) and is placed through an object relative to the object’s centre of mass to yield stable grasping. Van Bergen et al. [21], refined the concept of the ‘opposition axis’ and suggested it should be conceptualised as an ‘opposition vector’. The maximum magnitude of the opposition vector is a function of hand size which is why Mon-Williams and Bingham [16] related this factor to the task safety margins. But whilst hand anatomy sets an absolute limit on the maximum magnitude, maximum grasp aperture is not a simple function of hand size. Maximum grasp aperture is additionally a function of the extent to which an individual has the ability to use their anatomical span to position their digits to obtain a stable grasp. Thus, a reduction in joint flexibility (for example) will decrease the available range of positional adjustments and thereby diminish the safety margins when grasping a given object.

The preceding consideration suggests that the safety margins of the task will be related to factors such as the flexibility of the finger joints. The present study compares the reach-to-grasp actions of older participants with those of younger participants. We hypothesised that physiological alterations in the hand associated with older age (reduced joint flexibility) would produce changes in reach-to-grasp movements. Standard kinematic measures were taken while object size, object position and object texture were varied. We were particularly interested in the impact of a rougher (higher friction) texture since this object property could be used to improve the design of objects so that they are better suited to an older population (as determined on the basis of empirical measures).

## Methods

Two groups of twelve unpaid participants were recruited, an older group (9 female; age mean 73.5 years, age range 62.1–84.0 years; 11 reported right hand preference) and a younger group (7 female; age mean 27.7 years, age range 20.5–47.1 years; 11 reported right hand preference). The older adults were active members in the Yorkshire and Humber region of the University of the Third Age. As such the participants were physically active, indepently living and in continuing education. All participants had normal or corrected-to-normal vision and no history of neurological deficit. Maximum pinch grip aperture was measured for each participant (for the older group, mean 14.6 cm, range 13.1–16.5 cm; for the younger group, mean 15.8 cm, range 13.0–21.0 cm). All participants provided written informed consent prior to inclusion in the study. The study was approved by the University of Leeds IPS ethics committee and was performed in accordance with the ethical standards laid down in the Declaration of Helsinki.

Participants were asked which hand was their preferred hand (indexed by hand used for writing and throwing a ball). With their preferred hand, participants were asked to reach-grasp-and-lift specially manufactured objects: plastic (black nylon) cylinders (25.4mm diameter) mounted on wooden blocks (the size of the mounting block was proportional to the cylinder length). The ends of each plastic cylinder were machined to a 25mm radius. Participants grasped the curved end faces of the cylinder between the thumb and index finger thus the three lengths of the cylinder (5, 7 and 9 cm) represented the narrow, medium and wide stimulus “widths” respectively. For each of the three stimulus widths, there were two different finishes applied to the grasp surfaces such that two distinct coefficients of friction would be generated: High (μ_H_), and Low (μ_L_). The high-friction surface (μ_H_) was generated by sticking coarse-grade sandpaper (Aluminium Oxide, P50) to the grasp surfaces. The low-friction condition (μ_L_) was achieved through the application of petroleum jelly (Vaseline®, Unilever) with a soft-bristled brush to the participants’ fingertips and the grasp surfaces of the stimulus between trials of this condition (application was repeated on alternate trials).

To ensure a consistent starting position the participants pinched a raised origin marker positioned 10 cm from the front edge of the study table prior to the start of each trial. The objects were placed at distances of 10, 30 and 50 cm beyond the origin point in line with the midline of the participant. Participants were instructed to reach and grasp the object as quickly and as accurately as possible between the pads of the forefinger and thumb, lift the stimulus from the table and hold it in a static raised position until told to lower the object to the table and return to the start position in preparation for the next trial.

The factors of object width and distance were altered in a pseudo-randomised order. Trials were blocked and counterbalanced on the factor of surface friction coefficient. The three object widths, three object distances and two coefficients of friction represented 18 conditions, each of which was repeated 10 times resulting in a total of 180 trials. The test session typically lasted 45 minutes. Trial repetition criteria included: (i) failure to grip the stimuli on the instructed surface; (ii) inability to achieve stable, static grip of the stimuli; (iii) knocking the stimuli over (iv) dropping the object prior to, or shortly after the verbal return command. Following failure of a trial, the condition under which failure occurred was recorded and the participant returned to the origin and repeated the test. For multiple trial failures, this was repeated until 10 trials for each condition were complete. The degree of handedness was not included in the analysis.

**Figure 1:**
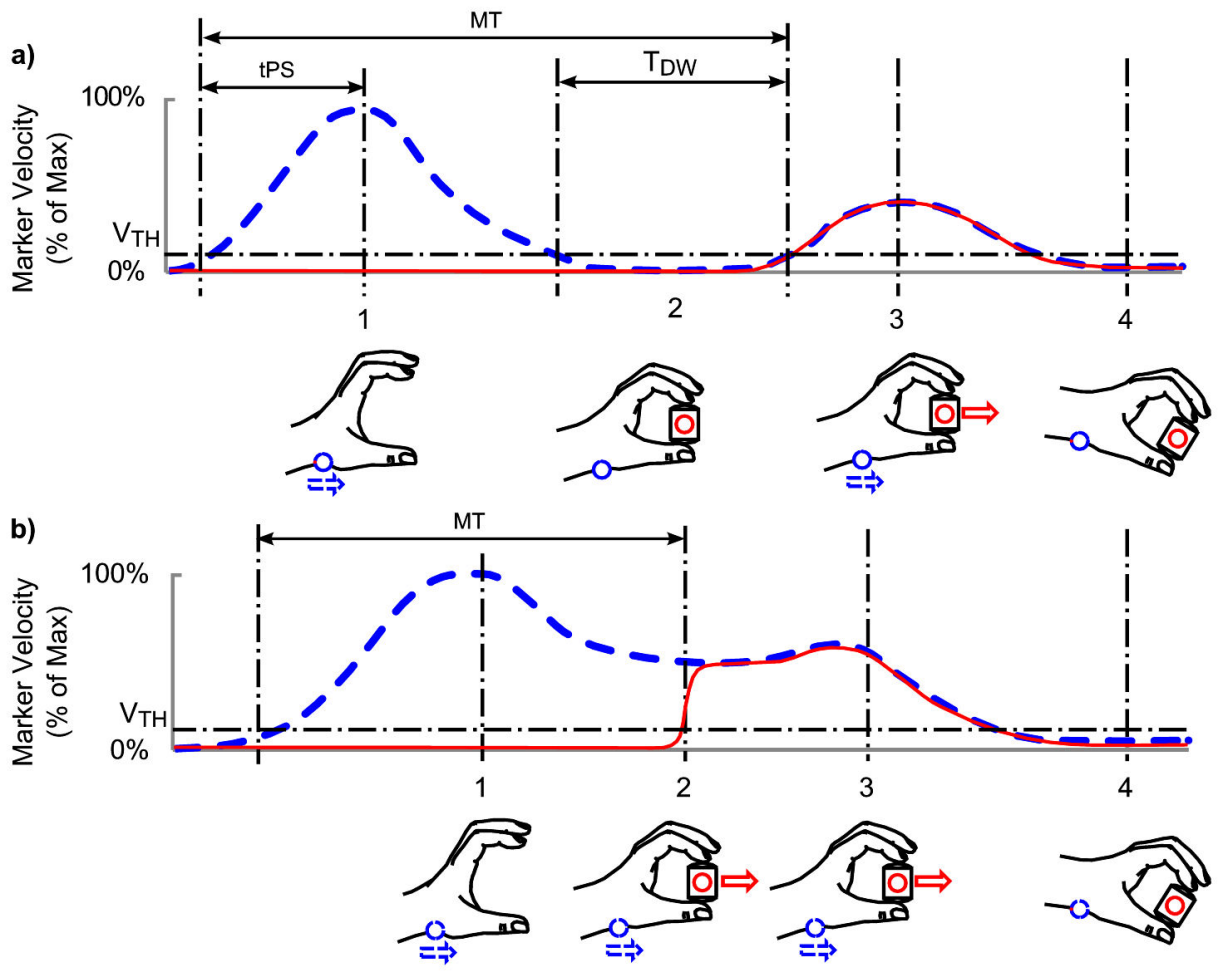
Kinematic profiles for stop and ‘on-the-fly’ prehension movements. *Upper* A velocity profile typical of a stop movement: 1, the hand is in the transport phase with the wrist IRED reaching peak velocity. 2, as the hand and fingers approach the object the hand velocity drops below the threshold velocity (Vth) and remains below threshold velocity or stops for a period (T_DW_). 3, upon successful application of the grip, both the wrist and object markers move in unison as part of a second distinct movement. 4, movement complete – hand and object velocity tends to zero. Time to Peak Speed (tPS) is defined as the time between the wrist marker moving above Vth and achieving peak speed. Movement time is defined as the time elapsed between the wrist marker achieving Vth and the object marker achieving Vth, here represented in the stop movement scenario. *Lower* A velocity profile typical of a ‘fly-through’ movement: 1, the hand is in transport phase toward the object. 2, as the fingers contact the object, the wrist IRED velocity is maintained above the threshold velocity (Vth) as the object is gripped. 3, the hand and object continue to move in unison while the wrist IRED velocity remains above the threshold velocity. 4, movement complete, hand and object velocity tends to zero. Movement time is defined as the time elapsed between the wrist marker achieving Vth and the object marker achieving Vth, here represented in the fly-through movement scenario.

Kinematic data acquisition was performed using an Optotrak 3020 motion tracking system (Northern Digital, Ontario, Canada). The positions of four Infra Red Emitting Diodes (IREDs) were acquired at 100Hz for four seconds for the μ_H_ and for five seconds on the μ_L_ conditions (because the reduced friction surface took longer to pick up). The first two markers were attached to the reaching hand at the index finger (distal medial corner of the finger) and the thumb (distal lateral corner of the thumb). These markers were used to measure grip aperture. The third marker was placed on the styloid process of the wrist to provide an independent measure of hand movement. A fourth marker was placed on the wooden block of the stimulus to identify when the object was lifted off the tabletop. All data were filtered using a dual-pass Butterworth second order filter with a cut-off frequency of 16Hz (equivalent to a fourth order zero phase lag filter of 10Hz). The speed of the wrist IRED and the aperture was computed and the onset and offset of movement was estimated using a standard algorithm (threshold for movement onset and offset was 5 cm/s as per Munro, Plumb, Wilson, Williams and Mon-Williams [22]). Custom analysis routines were used to compute the dependent variables of interest in this study (Figure 1). A wrist marker velocity raising above and falling below 5 cm/s was used as the threshold speed defining the onset and offset of movements respectively. Similarly, the criterion for onset and termination of the grasp closure was when relative finger speed (aperture open/closure rate) dropped above and below 5 cm/s respectively. The object’s ‘time-to-lift’ was designated at the point when the fourth IRED’s velocity exceeded 5 cm/s. We designated movements as ‘stop’ when the wrist stopped moving prior to movement of the object and ‘fly through’ when wrist movement stopped after the object was lifted (see Figure 1). In stop trials we calculated the ‘dwell time’ which was defined as the temporal difference between the wrist stopping forward movement and the object being lifted. Movement time was defined as the duration between the onset of wrist movement and the onset of the object moving. Time to peak speed (tPS) was defined as the elapsed time in the pre-contact movement from the wrist reaching threshold speed and the time taken to reach peak speed. To ensure that any age differences in tPS were not purely a function of movement duration, tPS was normalised against movement duration to obtain normalised time to Peak Speed (ntPS) calculated as the ratio of total pre-contact movement time to time to peak speed.

**Figure 2:**
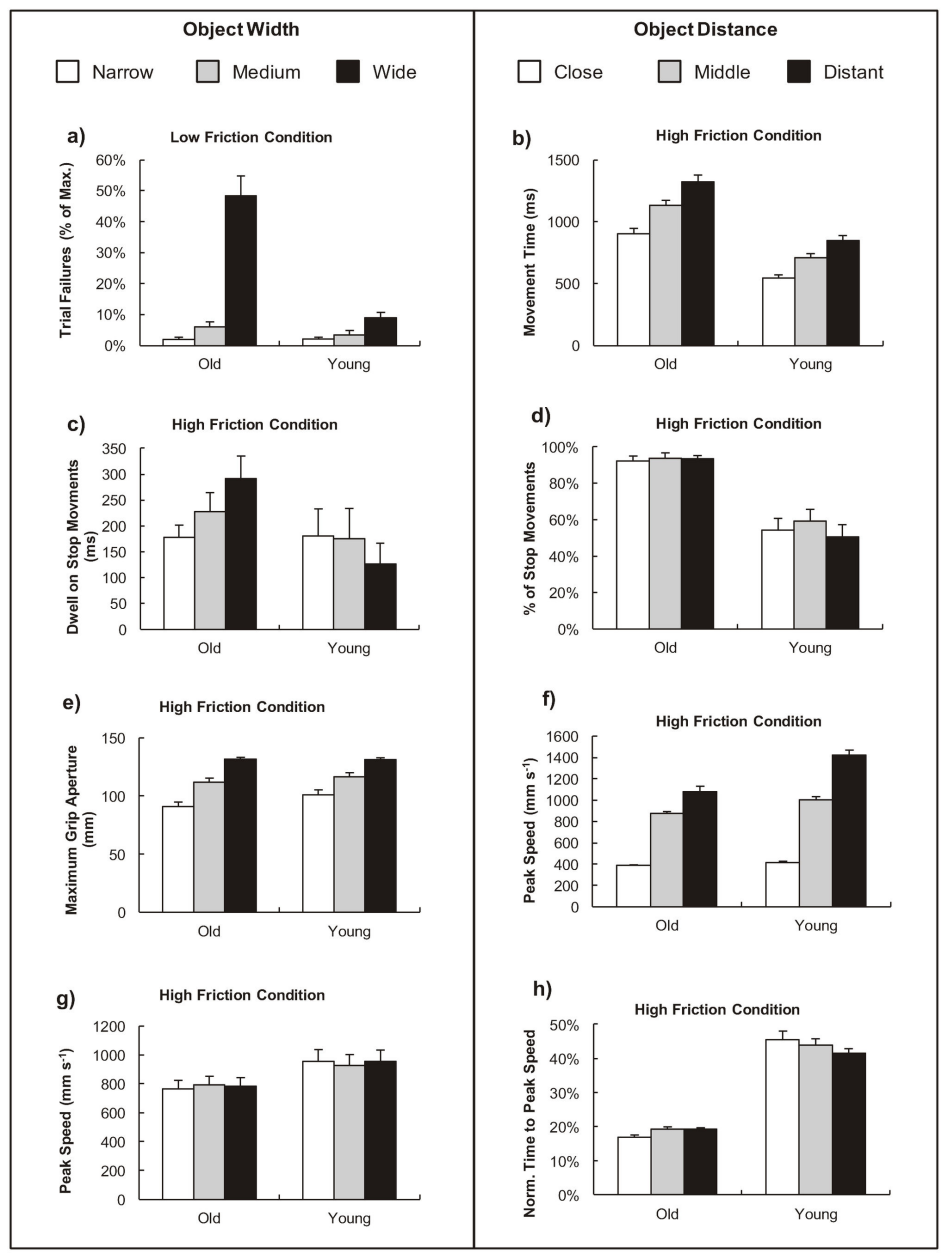
Kinematic measures for two age groups grasping objects of varied texture. Comparisons of kinematic measures where key significant differences were found between older and younger groups as a function of object width (left panel) and object distance (right panel).

All participants managed to grasp-and-lift the objects with the high friction coefficients most of the time (mean failure across both groups and all widths for the high friction coefficient was 0.1%). In contrast, the low friction condition caused greater difficulties and the percentage of failed trials varied as a function of condition and group (Figure 2a) so we explored this statistically. Because we had no complete kinematic data for failed trails, we restricted our kinematic comparisons between the groups to the high friction condition. Comparisons were made on six kinematic measures: peak speed; time to peak speed; maximum grip aperture; proportion of ‘stop’ movements; dwell time; and movement time. The average value (across trials) was calculated for each participant for each condition, and a separate mixed ANOVA (distance (3) x object width (3) x age (2)) was carried out on each kinematic measure. Partial eta squared (η_p_^2^) values are reported for statistically significant findings. The data were tested for violations of sphericity and, where the assumption of sphericity was not met, Greenhouse-Geisser corrections of epsilon (ε) were applied to the degrees of freedom.

## Results

### Low Friction objects: Proportion of Trial Failures

The number of dropped slippery objects was calculated as a percentage of the maximum allowed drops before the condition was abandoned (max. 15 attempts). There was a significant object width x age interaction (F(2,44) = 13.930, p < 0.01, ε = 0.537, η_p_^2^ = 0.388; Figure 2a) with the wide slippery object causing the older adults disproportionate difficulty. There were no other interactions.

### High Friction objects: Movement Time (MT)

There was a significant main effect of distance (F(2,44) = 114.19, p < 0.001, ε = 0.663, η_p_^2^ = 0.838) (Figure 2b), and a significant main effect of object width (F(2,44) = 7.146, p < 0.01, η_p_^2^ = 0.245). There was a significant effect of age (F(1,22) = 25.48, p < 0.001, η_p_^2^ = 0.537) with increased MT for older people, but no significant interactions.

### High Friction objects: Proportion of ‘stop’ movements and dwell time

A higher proportion of reaches made by older adults stopped moving prior to lifting compared to the young (F(1,22) = 13.025, p < 0.01, η_p_^2^ = 0.372; Figure 2d), but this did not change across distance (F(2,42) = 0.77, p = 0.43, ε = 0.76) or width (F(2,42) = 1.18, p = 0.32). For those reaches that did stop, there was no effect on dwell time of age, distance or object width. There was an age x width interaction (F(2,34) = 4.096, p < 0.05, ε = 0.714; Figure 2c) which reflects older adults taking longer to adjust hand position around wider objects.

### High Friction objects: Maximum Grip Aperture (MGA)

There was an interaction between object width and participant age on maximum grip aperture (F(2,44) = 3.88, p < 0.05, η_p_^2^ = 0.15; Figure 2e). Inspection of the results showed that the interaction was driven by all participants scaling their grasp aperture to the object widths but the younger participants showing a larger MGA for the narrow object. This can be explained by the faster approach speed of the younger participants requiring a greater safety margin (provided by a larger aperture). There was also an interaction between object width and reach distance on maximum grip aperture; (F(4,88) = 3.22, p < 0.05, η_p_^2^ = 0.128). This was caused by a decreased aperture for the narrow objects when positioned further away but no effect of distance with the wider objects. The combined effects described between width and distance and width and age combined to produce a three way interaction (F(4,88) = 5.72, p < 0.01, η_p_^2^ = 0.206).

### High Friction objects: Peak Speed (PS) and normalised time to Peak Speed (ntPS)

Peak speed (PS) showed a significant object distance × age interaction that reflects the reduced scaling of movement speed by older adults to more distant objects (F(2,42) = 6.969, p < 0.01, η_p_^2^ = 0.574; Figure 2f). There was a significant interaction between object width and age (F(2,44) = 3.996, p < 0.05, η_p_^2^ = 0.154; Figure 2g). While there were no systematic differences between PS across object widths, the young reduced PS slightly for medium width objects, whereas older adults increased PS for medium width objects. There was no significant three way interaction.

We examined the time at which peak speed occurred during the reach movement. There was a significant interaction between distance and age (F(2,44) = 6.069, p < 0.05, ε = 0.639, η_p_^2^ = 0.216; Figure 2h). Younger adults reached peak speed much later in the movement (approximately half way through the movement). They also scaled their movements so that peak speed occurred earlier for further distances, in contrast to older adults who kept ntPS similar across object distance. There was no significant main effect of object width or other interactions.

## Discussion

Older adults showed particular difficulties when reaching-to-grasp and lift wide slippery objects. The older participants were high functioning individuals (e.g. emeritus professors) who appeared to have no physical ailments (including arthritis) and no impairments in excess of ‘normal’ age related changes. Thus, our results show that reaching-to-grasp objects poses a major challenge to even the healthy and ‘successfully aging’ proportion of the older population. The difficulties observed in these adults are likely to be greater in older adults with arthritis or neurological deficits (e.g. stroke, Parkinson’s disease etc). The centrality of prehensile behavior to many activities of daily living suggests that a priority area within human factors research should be to understand how objects can be engineered to make them easier to handle for older adults. In our experiment, the failure to lift the objects had no practical consequences. In contrast, an object slipping out of the hand can have disastrous consequences outside the laboratory (e.g. dropping a pan after a handle has become greasy). It follows that our findings raise major issues relating to safety for older adults.

A kinematic investigation of the reach-to-grasp movements to high friction objects (movements which were generally successful) revealed both quantitative and qualitative changes as a function of age. The older group generally adopted a more cautious strategy, using slower movements, with peak speed reaching a maximum earlier in the movement and so a longer deceleration phase before grasping the object. The older participants also exhibited fewer “fly-through” movements, consistent with cautious grasping. Most notably, the older group showed less evidence of tailoring their reach and grasp to object distance and width than the younger group. The older adults showed little change in time to peak speed for different stimuli distances, whereas the young scaled their movements appropriately.

Significantly, the older group had far greater problems than the younger group with picking up the widest object in the low friction condition. Our findings seem best explained by a simple lack of an ability to adjust finger position precisely in older individuals. The rough objects provided a large effective zone for stable grasping whereas the slippery objects required precise positioning of the digits in the centre of the grasping surface [17]. Notably these results could not be predicted by a simple anatomical examination of the hand (i.e. simply measuring maximum grip aperture) – rather it was a functional inability to enclose the object that caused the behavioural difficulties.

The findings from the present experiment fit well within the theoretical framework of ‘affordance perception’ outlined in the introduction. An analysis of reach-to-grasp movements shows that collision avoidance is a central component of the task [10]. Successful grasping (i.e. grasping without knocking an object over) requires that the aperture between the finger(s) and thumb be opened wide enough to avoid hitting the object before the digits can enclose the object. In the current experiments we anticipated that the safety margins would be scaled to the relevant body dimension and we hypothesised that the maximum functional grasp span would be the relevant body dimension as this determines the available safety margin. Nevertheless, functional action adaptations to task constraints are well documented [23] and other task variations are known to alter the structure of reaches-to-grasp [24]. For example, increasing the speed of the reach will cause greater variability of the sizing, positioning and/or orienting of the opposition axis. It follows that decreasing the speed of the reaches can compensate for lower safety margins created by a reduced maximum functional grasp span. This might explain why the older participants showed slower reach-to-grasp movements - a behavioural change consistent with reports that older adults make strategic compensations in order to achieve success within a visual-motor task [25].

The most parsimonious explanation for our findings is reduced positioning control in the hand of the older adults. Nevertheless, other motor and cognitive factors might have contributed towards the difficulties the older adults experienced when trying to grasp and lift the slippery objects [26,27]. More research is required to document the changes that occur in the hand as a function of age but an important implication of our findings is that performance cannot be predicted simply from anatomical measurements. Identifying the limits to reaching-to-grasp requires an examination of success or failure in functional tasks. The present study suggests that kinematic measures can provide a powerful tool for the purpose of examining performance in functional tasks and thereby evaluate how different product designs can support safer grasping in older adults.

## References

[1] Nouri FM, Lincoln NB (1987) An extended activities of daily living scale for stroke patients. Clin Rehabil 1: 233-238.

[2] Mahoney FI, Barthel DW (1965) Functional Evaluation: The Barthel Index. MD State Med J 14: 61-65. PubMed: 14258950. 14258950

[3] Metcalfe CD, Woodward H, Wright V, Chappell PH, Burridge JH et al. (2008) Changes in Hand Function with Age and Normative Unimpaired Scores when Measured with the Southampton Hand Assessment Procedure. Hand Therapy 13: 79-83.

[4] Hackel ME, Wolfe GA, Bang SM, Canfield JS (1992) Changes in hand function in the aging adult as determined by the Jebsen Test of Hand Function. Phys Ther 72: 373-377. PubMed: 1631206. 1631206 10.1093/ptj/72.5.373

[5] Marmon AR, Pascoe MA, Schwartz RS, Enoka RM (2011) Associations among strength, steadiness, and hand function across the adult life span. Med Sci Sports Exerc 43: 560-567. doi:10.1249/01.MSS.0000401544.85004.01. PubMed: 20689447. 20689447 10.1249/MSS.0b013e3181f3f3ab

[6] Frederiksen H, Hjelmborg J, Mortensen J, McGue M, Vaupel JW et al. (2006) Age trajectories of grip strength: cross-sectional and longitudinal data among 8,342 Danes aged 46 to 102. Ann Epidemiol 16: 554-562. doi:10.1016/j.annepidem.2005.10.006. PubMed: 16406245. 16406245 10.1016/j.annepidem.2005.10.006

[7] Shechtman O, Mann WC, Justiss MD, Tomita M (2004) Grip strength in the frail elderly. Am J Phys Med Rehabil 83: 819-826. doi:10.1097/01.PHM.0000143398.00788.4E. PubMed: 15502734. 15502734 10.1097/01.phm.0000143398.00788.4e

[8] Gilles MA, Wing AM (2003) Age-related changes in grip force and dynamics of hand movement. J Mot Behav 35: 79-85. doi:10.1080/00222890309602123. PubMed: 12724101. 12724101 10.1080/00222890309602123

[9] Bootsma RJ, Marteniuk RG, MacKenzie CL, Zaal FT (1994) The speed-accuracy trade-off in manual prehension: effects of movement amplitude, object size and object width on kinematic characteristics. Exp Brain Res 98: 535-541. PubMed: 8056073. 8056073 10.1007/BF00233990

[10] Rosenbaum DA, Meulenbroek RG, Vaughan J, Jansen C (1999) Coordination of reaching and grasping by capitalizing on obstacle avoidance and other constraints. Exp Brain Res 128: 92-100. doi:10.1007/s002210050823. PubMed: 10473746. 10473746 10.1007/s002210050823

[11] Napier JR (1956) The prehensile movements of the human hand. J Bone Joint Surg Br 38-B: 902-913. PubMed: 13376678. 13376678 10.1302/0301-620X.38B4.902

[12] Mon-Williams M, Tresilian JR (2001) A simple rule of thumb for elegant prehension. Curr Biol 11: 1058-1061. doi:10.1016/S0960-9822(01)00293-7. PubMed: 11470412. 11470412 10.1016/s0960-9822(01)00293-7

[13] Smeets JB, Brenner E (1999) A new view on grasping. Mot Contr 3: 237-271. 10.1123/mcj.3.3.237 10409797

[14] Jeannerod M (1984) The timing of natural prehension movements. J Mot Behav 16: 235-254. PubMed: 15151851. 15151851 10.1080/00222895.1984.10735319

[15] Jeannerod M (1988) The neural and behavioural organization of goal-directed movements. Oxford: Oxford University Press.

[16] Mon-Williams M, Bingham GP (2011) Discovering affordances that determine the spatial structure of reach-to-grasp movements. Exp Brain Res 211: 145-160. doi:10.1007/s00221-011-2659-2. PubMed: 21484397. 21484397 10.1007/s00221-011-2659-2

[17] Flatters IJ, Otten L, Witvliet A, Henson B, Holt RJ et al. (2012) Predicting the effect of surface texture on the qualitative form of prehension. PLOS ONE 7: e32770. doi:10.1371/journal.pone.0032770. PubMed: 22403706. 22403706 10.1371/journal.pone.0032770 PMC3293844

[18] Gibson JJ (1977) The theory of affordances. In: Shaw R Perceiving, acting, and knowing: towards an ecological psychology. Hillsdale, NJ: Erlbaum.

[19] Turvey MT, Shaw RE, Reed ES, Mace WM (1981) Ecological laws of perceiving and acting: in reply to Fodor and Pylyshyn (1981). Cognition 9: 237-304. doi:10.3758/BF03196958. PubMed: 7197604. 7197604 10.1016/0010-0277(81)90002-0

[20] Iberall T, Bingham GP, Arbib MA (1986) Opposition space as a structure concept for the analysis of skilled hand movements. Exp Brain Res 15: 158-173.

[21] van Bergen E, van Swieten LM, Williams JH, Mon-Williams M (2007) The effect of orientation on prehension movement time. Exp Brain Res 178: 180-193. doi:10.1007/s00221-006-0722-1. PubMed: 17053908. 17053908 10.1007/s00221-006-0722-1

[22] Munro H, Plumb MS, Wilson AD, Williams JH, Mon-Williams M (2007) The effect of distance on reaction time in aiming movements. Exp Brain Res 183: 249-257. doi:10.1007/s00221-007-1040-y. PubMed: 17639361. 17639361 10.1007/s00221-007-1040-y

[23] Bril B, Rein R, Nonaka T, Wenban-Smith F, Dietrich G (2010) The Role of Expertise in Tool Use: Skill Differences in Functional Action Adaptations to Task Constraints. J Exp Psychol Hum Percept Perform 36: 825-839. doi:10.1037/a0018171. PubMed: 20695702. 20695702 10.1037/a0018171

[24] Marteniuk RG, Mackenzie CL, Jeannerod M, Athenes S, Dugas C (1987) Constraints on Human Arm Movement Trajectories. Can J Psychrev Can Psychol 41: 365-378. doi:10.1037/h0084157. PubMed: 3502905. 10.1037/h0084157 3502905

[25] Raw RK, Kountouriotis GK, Mon-Williams M, Wilkie RM (2012) Movement control in older adults: does old age mean middle of the road? J Exp Psychol Hum Percept Perform 38: 735-745. doi:10.1037/a0026568. PubMed: 22141585. 22141585 10.1037/a0026568

[26] van Swieten LM, van Bergen E, Williams JH, Wilson AD, Plumb MS et al. (2010) A test of motor (not executive) planning in developmental coordination disorder and autism. J Exp Psychol Hum Percept Perform 36: 493-499. doi:10.1037/a0017177. PubMed: 20364932. 20364932 10.1037/a0017177

[27] Christou EA, Enoka RM (2011) Aging and movement errors when lifting and lowering light loads. Age 33: 393-407. doi:10.1007/s11357-010-9190-4. PubMed: 20945163. 20945163 10.1007/s11357-010-9190-4 PMC3168598

